# The UK Paediatric Familial Hypercholesterolaemia Register: preliminary data

**DOI:** 10.1136/archdischild-2015-308570

**Published:** 2016-03-06

**Authors:** Uma Ramaswami, Jackie Cooper, Steve E Humphries

**Affiliations:** 1 Lysosomal Disorders Unit, Royal Free Hospital, London, UK; 2 British Heart Foundation Laboratories, Centre for Cardiovascular Genetics, Institute of Cardiovascular Science, the Rayne Building University College London, London, UK

**Keywords:** Genetics, Health services research, Paediatric Practice

## Abstract

**Background:**

The National Institute for Health and Care Excellence 2008 guidelines on the treatment and management of familial hypercholesterolaemia (FH) recommend that children with FH should be considered for statin treatment by the age of 10 years. The Paediatric FH Register was established in 2012 to collect baseline and long-term follow-up data on all children with FH in the UK.

**Methods:**

Paediatricians and adult lipidologists have been invited to enter baseline data on any child with a clinical diagnosis of FH using an electronic capture record.

**Results:**

Baseline data is on 232 children (50% boys, 80% Caucasian), with an untreated mean (SD) total cholesterol of 7.61 (1.48) mmol/L and low-density lipoprotein cholesterol (LDL-C) of 5.67 (1.46) mmol/L. Overall 111/232 (47.8%) of the children were on statins. Children over the age of 10 years at the most recent follow-up were twice as likely to be on statin treatment than those under 10 years (57.6% (102/177) vs 23.1% (9/39), p=0.00009). In both age groups, those subsequently on statin treatment had significantly higher diagnostic total and LDL-C (overall 6.01 (1.46) mmol/L vs 5.31 (1.37) mmol/L, p=0.00007), and had stronger evidence of a family history of early coronary heart disease (CHD) in parent or first-degree relative (overall 28.4% vs 19.0%, p=0.09). In statin-treated children LDL-C level was reduced by 35% (2.07 (1.38) mmol/L) compared with a reduction of 5.5% (0.29 (0.87) mmol/L), p=0.0001 in those not treated. None of those on statin had measured plasma levels of creatine kinase, alanine aminotransferase and AST indicative of statin toxicity (ie, >2.5 times the upper limit of the normal range).

**Conclusions:**

The data indicates that treatment decisions in children with FH are appropriately based on a stronger family history of CHD and higher LDL-C.

**What is already known on this topic**

- Children with a clinical diagnosis of familial hypercholesterolaemia (FH) have elevated levels of low-density lipoprotein cholesterol from birth, and elevated future risk of early coronary heart disease.
- CG71 recommends that all children with FH should be considered for treatment with a statin by the age of 10 years using clinical judgement.
- The National Institute for Health and Care Excellence (NICE) Guideline CG71 recommends all children with FH should have a DNA test to confirm their diagnosis.

**What this study adds**

- This is the largest group of UK children examined to date with a clinical diagnosis of familial hypercholesterolaemia (FH).
- Children less than 10 years of age and adolescents were more likely to be on statins in the presence of a family history of early heart disease and higher low-density lipoprotein cholesterol.
- In only 64% of children the family mutation was known.

## Background

Familial hypercholesterolaemia (FH) is an autosomal dominant inherited disorder characterised by elevated low-density lipoprotein cholesterol (LDL-C) levels from birth.^1^ The prevalence of heterozygous FH (HeFH) is thought to be 1 in 500, although recent studies have indicated the prevalence may be twice as high.^2^ ^3^ HeFH is therefore estimated to affect 20–40 million people worldwide. In patients with HeFH, premature coronary heart disease (CHD) manifests in about half of men by age 50 years and a third of women by age 60 years,^4^ however, the vast majority of patients with HeFH remain undiagnosed. In the UK, based on a prevalence of 1:500 and the known population age structure, ∼14 000 children under the age of 10 years will have HeFH, with an additional 14 000 between the age of 10–18 years. The 2010 UK National FH audit data suggested that less than 800 of these have been identified and are being clinically managed.^5^

In the UK the 2008 National Institute for Health and Care Excellence (NICE) FH guideline (CG71) recommended the use of the Simon Broome criteria for diagnosing children with FH,^6^ using a total cholesterol (TC) of >6.7 mmol/L and an LDL-C of >4.0 mmol/L, together with a family history of elevated TC and premature CHD. In adults with a personal or family history of tendon xanthomas (TX) a diagnosis of definite FH (DFH) is given, while if TX are absent a diagnosis of possible FH (PFH) is given.^4^ Since TX usually develop only in the third decade of life the DFH/PFH distinction is not used for children. The plasma total and LDL-C levels that are used as diagnostic criteria for FH probands in the general population are too stringent for use in relatives, given the higher prior probability of a first-degree relative having FH (50% vs 1/500). To overcome this, sex-specific LDL-C diagnostic cut-offs were established^7^ designed to give the greatest overall accuracy when testing relatives of patients with FH in the absence of a genetic diagnosis, and their use was recommended by NICE CG71. However, mutation testing is recommended as the unambiguous ideal cascade screening test for FH.

In adults with FH, an underlying genetic cause for the disorder can be identified in ∼80% of DFH cases,^8^ and is most often attributable to mutations within the *LDLR* gene which encodes the low-density lipoprotein receptor. Mutations in apolipoprotein B and proprotein convertase subtilisin/kexin type 9 can produce phenotypes identical to *LDLR* FH.^9^ In the majority of patients where no causative mutation can be found a polygenic cause of their hyperlipidaemia is most likely.^10^ ^11^

Over the last 5 years several countries have produced guidelines for the identification and management of adults and children with FH.^1^ ^12–15^ All of them recommend the use of lipid-lowering statin therapy in children, but the age at which statin use should be started, or its intensity, to best prevent the onset of adult premature CHD has not been established, since there are no long-term randomised controlled outcome trials for ethical reasons. By contrast there is considerable short-term randomised and observational data available on the management of children with HeFH, showing a good safety profile, without liver toxicity side effects, no influence on growth trajectory and excellent efficacy in terms of LDL-C reduction over periods of 2–3 years.^16^ ^17^

**Figure 1:**
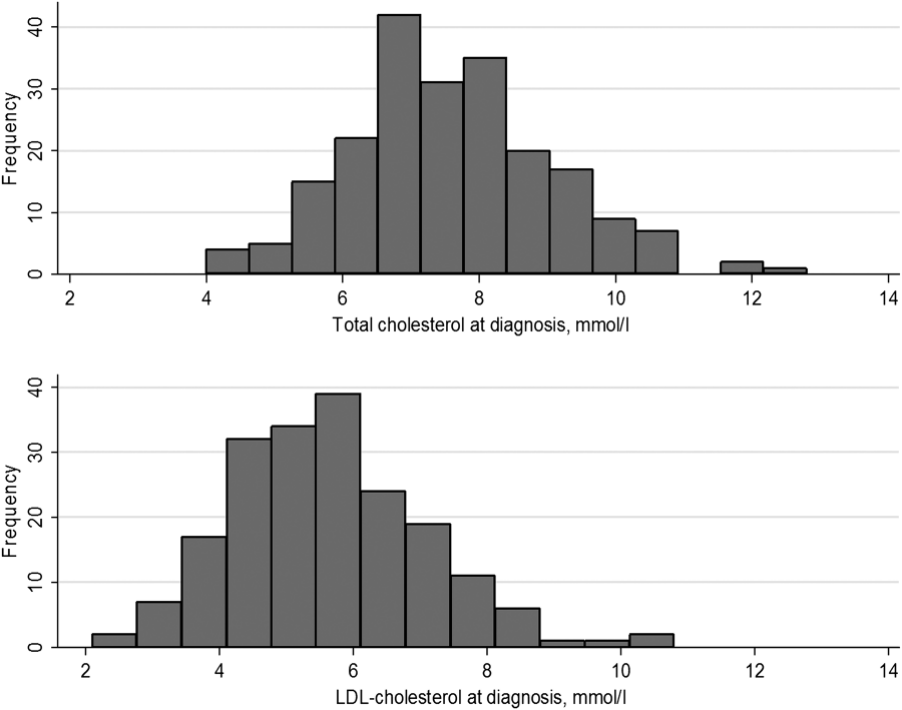
Histogram of the measured total cholesterol and low-density lipoprotein cholesterol (LDL-C) in the children with heterozygous familial hypercholesterolaemia (HeFH).

Children with HeFH have roughly twice the normal LDL-C levels from birth and thus their LDL-C burden (average level×years of age) increases at twice the rate of their non-FH siblings^16^ (see figure 1). As a consequence of this they develop atherosclerosis that is detectable as significant carotid intima media thickness (CIMT) as compared with their siblings by the age of 10 years.^18^ ^19^ In a randomised controlled trial of the use of pravastatin, further change in CIMT was prevented.^20^ Based on this data the NICE guidance is that the use of statins should be considered in children with HeFH by the age of 10 years using clinical judgement, based on the child's LDL-C level, the age of onset of CHD in the parent or relatives, and the presence of other CHD risk factors.

The National Paediatric FH Register was established in 2012. The aim of this register is to collect baseline and long-term follow-up data on all children with FH in UK, to document how well current NICE guidance on the diagnosis and management of children with HeFH is being adhered to, and to determine the safety and efficacy of statins commenced below the age of 16 years. We report here the preliminary results on the first 232 registered children.

## Methods

The register steering committee includes a patient representative, adult and child physicians and representatives of Heart UK, British Heart Foundation, British Inherited Metabolic Disease Group, and the Royal College of Paediatrics and Child Health. An electronic web based data capture tool was developed to provide comparative audit and long-term safety data, and anonymised data for research (see online supplementary appendix 1). The Royal College of Physicians (RCP) currently acts as the host server for the register. All lipid clinics in England and paediatricians with an interest in lipid disorders were contacted and details of the register provided. To enrol patients each centre was provided with a centre-specific code and password.

10.1136/archdischild-2015-308570.supp1 Supplementary appendix

### Statistical methods

Results for continuous variables are presented as mean (±SD) and median (with IQR), and differences by sex and statin use are tested using Mann-Whitney U tests. Differences in height, weight and the fall in LDL-C by statin use are adjusted for age using analysis of covariance. Categorical variables are presented as percentages and number, and tested using χ^2^ tests or Fisher's exact test. Changes in LDL-C by statin use were analysed using analysis of covariance with adjustment for age and length of follow-up. Differences in the effect of statin by age group were tested by including an interaction term in the model. Cox proportional hazards models are used to take account of the length of follow-up as well as age at diagnosis when looking at time to statin commencement. Statin-induced toxicity was examined as the proportion of children having levels over 2.5×the upper limit of normal as described in the Common Terminologies Criteria for Adverse Events guidelines (see online supplementary table S4 for details).

## Results

Data was analysed from July 2012 to November 2014. Twenty-eight centres have thus far enrolled patients (see online supplementary appendix 2). Recruitment rates vary from 1 to 29 patients per centre (median 5 patients). The majority of patients have a diagnosis of HeFH (98.7% (232/235)), while 3 individuals had a TC >15 mmol/L compatible with a diagnosis of homozygous FH, and were excluded from the analysis. The majority were white Caucasian (n=185; 78.7%), 10.6% were of Indian subcontinent origin (n=25). Only four of the individuals (1.7%) were reported as cigarette smokers.

Since NICE CG71 recommends testing of children at risk of HeFH by the age of 10 years, we present the characteristics of the registered children divided into those below and above this age (table 1). Of the 232 HeFH children registered, there are equal numbers of boys and girls (50% boys). Overall, the untreated mean (±SD) TC was 7.61(1.48) mmol/L and LDL-C of 5.67 (1.46) mmol/L (figure 1), with levels of high-density lipoprotein cholesterol (HDL-C) and triglycerides (TG) within the normal range. Three of the children had a TG level >3.5 mmol/L but this had fallen to below 2.0 mmol/L at follow-up suggesting that initial levels may not have been from a fasting blood sample.

**Table 1:** Paediatric FH Register characteristics* at baseline by age

		≤10 years N=96†	>10 years N=111†
Number boys/girls (%age boys)		50/46 (52.1)	54/57 (48.7)
Age (years)	Mean (SD) N Median (IQR)	7.3 (2.2) 96 7.8 (5.5–9.1)	12.6 (1.7) 111 12.6 (10.9–13.9)
Weight (kg)	Mean (SD) N Median (IQR)	27.8 (9.5) 88 25.6 (21–34.1)	50.3 (14.4) 102 49.1 (40.5–57.7)
Height (m)	Mean (SD) N Median (IQR)	1.26 (14.2) 77 1.28 (1.17–1.37)	1.56 (11.4) 91 1.55 (1.49–1.64)
Total cholesterol (mmol/L)	Mean (SD) N Median (IQR)	8.07 (1.43) 96 8.0 (6.99–9.15)	7.22 (1.43) 111‡ 7.1 (6.3–8.0)
LDL-C (mmol/L)	Mean (SD) N Median (IQR)	6.17 (1.40) 87 6.1 (5.1–7.1)	5.28 (1.39) 106§ 5.1 (4.4–6.1)
HDL-C (mmol/L)	Mean (SD) N Median (IQR)	1.41 (0.35) 87 1.4 (1.13–1.65)	1.41 (0.33) 105 1.4 (1.2–1.6)
Triglyceride (mmol/L)	Mean (SD) N Median (IQR)	0.96 (0.47) 86 0.84 (0.7–1.1)	1.04 (0.60) 103 0.90 (0.67–1.25)
Smoking	% (N)	1.04% (1/96)	2.7% (3/110)

There are 31 adolescent girls on statins. Five do not have oral contraceptive use recorded. Four of the remaining 26 (15%) were prescribed oral contraceptives.

*Excluding 3 patients with homozygous FH.

†25 children are missing diagnosis dates.

‡Cholesterol is significantly lower in age>10 years versus age<10 years, p=0.00006.

§LDL-C is significantly lower in age>10 years vs age<10 years, p=0.00001

FH, familial hypercholesterolaemia; HDL-C, high-density lipoprotein cholesterol; LDL-C, low-density lipoprotein cholesterol.

We first examined to what extent the registered children fulfilled the NICE recommended LDL-C cut-off criteria.^7^ Only 2.6% of the children had LDL-C below the age-recommended and gender-recommended diagnostic cut-off for HeFH, with an additional 2.1% having levels in the ‘grey’ zone of undetermined FH status. Thus at diagnosis 95.3% had LDL-C levels above the age-adjusted and sex-adjusted cut-offs for FH status. This did not differ significantly between boys and girls (not shown).

**Figure 2:**
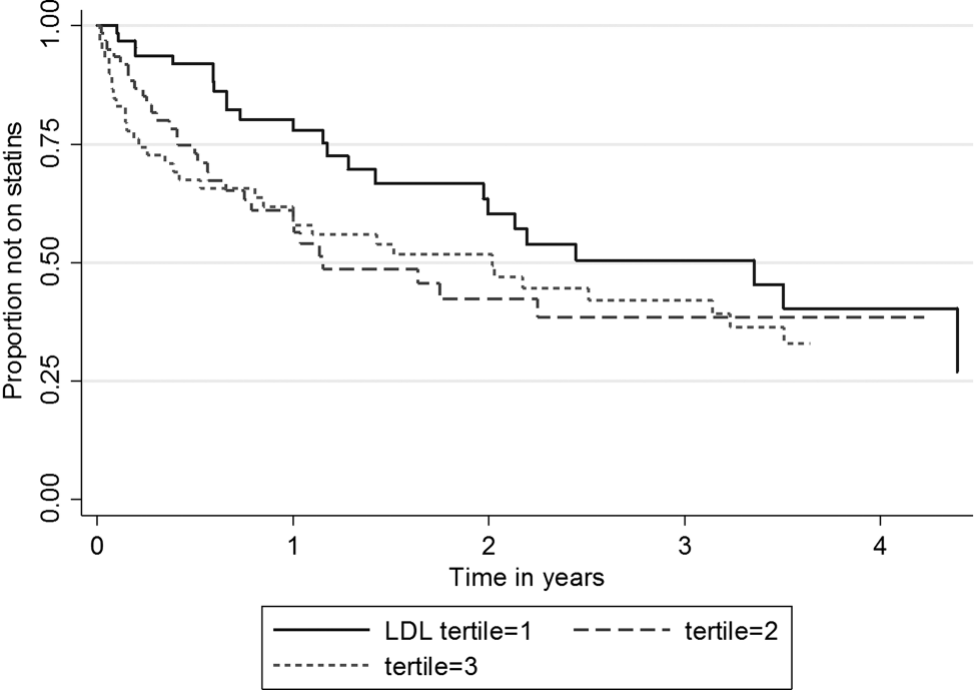
Kaplan-Meier plot for being treated with statin by tertile of baseline low-density lipoprotein cholesterol (LDL-C). Tertile cut-offs were Tertile 1 ≤4.9 mmol/L, Tertile 2 >4.9 and ≤6.1 mmol/l, Tertile 3 >6.1 mmol/L.

We next examined the differences in baseline characteristics between those who were subsequently prescribed statin at a follow-up visit at the last data cut-off for analyses (November 2014). Interestingly, table 1 and (figure 2) shows that the mean TC and LDL-C levels were significantly *lower* at registration in the children above 10 years, with (see online supplementary figure S1) a significant negative correlation between age and TC (R=−0.30, p=8.4×10^−06^) and age and LDL-C levels (R=−0.34, p=1.4×10^−06^). One possible explanation for this is that the younger children are more likely to be referred in light of a family history of early onset of CHD or a higher plasma level of LDL-C at diagnosis, while adolescents with a family history of FH are more likely to be referred to a lipid clinic regardless of their TC or LDL-C level.

Overall 111/232 (47.8%) of the children were on statins. Children over the age of 10 years at the most recent follow-up were twice as likely to be on statin treatment than those under 10 years (57.6% (102/177) vs 23.1% (9/39), p=0.00009). There were no significant differences in gender by statin use and girls were commenced on statins at a median age of 11.8 years compared with 11.2 years in boys (p=0.91). In those who subsequently were started on statin treatment, there was non-significant evidence in both age groups of a greater family history of early CHD in a parent or first-degree relative and, in the sample as a whole, family history of CHD in any relative was significantly more common in those subsequently started on statin treatment, with 48.7% (54/111) of those on statins having a family history in any relative compared with 33.9% (41/121) of those not on statins (p=0.02). In children where the family mutation was recorded as known or not known, there was no difference in the proportion with subsequent statin use (table 2).

**Table 2:** Characteristics at diagnosis by age at diagnosis and subsequent statin use

Age ≤10 years		Statin use		p Value (Mann-Whitney)
		No N=51	Yes N=45	
Age (years)	Mean (SD) Median (IQR)	7.4 (2.0) 51 7.7 (5.6–8.9)	7.1 (2.5) 45 7.8 (5.3–9.3)	0.84
Sex	% male	47.1% (24/51)	57.8% (26/45)	0.29 (χ^2^)
Ethnicity	% Caucasian	78.4 (40/51)	84.4 (38/45)	0.45 (χ^2^)
CHD in parent/first-degree relative	% yes	15.7% (8/51)	22.7% (10/44)	0.38(χ^2^)
CHD in any relative	%yes	33.3% (17/51)	48.9% (22/45)	0.12 (χ^2^)
CHD onset age in relative	Mean (SD) Median (IQR)	33.9 (20.5) 17 40 (30–49)	33.7 (16.8) 22 40 (23–44)	0.68
Mutation status	% yes	58.7% (27/46)	60.5% (26/43)	0.87 (chi-square)
Diag weight (kg)	Mean (SD) Median (IQR)	27.6 (9.5) 48 24.6 (21.4–32.5)	27.9 (9.6) 40 27.4 (20.4–34.4)	0.85
Diag height (m)	Mean (SD) Median (IQR)	1.25 (0.14) 43 1.27 (1.17–1.36)	1.27 (0.14) 34 1.29 (1.20–1.38)	0.64
Diag cholesterol (mmol/L)	Mean (SD) Median (IQR)	7.77 (1.42) 51 7.6 (6.8–8.4)	8.40 (1.39) 45 8.5 (7.3–9.3)	0.01
Diag HDL-C (mmol/L)	Mean (SD) Median (IQR)	1.44 (0.38) 47 1.4 (1.13–1.7)	1.37 (0.31) 40 1.39 (1.13–1.59)	0.62
Diag triglyceride (mmol/L)	Mean (SD) Median (IQR)	0.94 (0.54) 46 0.8 (0.64–1.1)	0.99 (0.39) 40 0.9 (0.76–1.18)	0.12
Diag LDL-C (mmol/L)	Mean (SD) Median (IQR)	5.83 (1.30) 48 5.8 (4.85–6.42)	6.59 (1.42) 39 6.7 (5.5–7.7)	0.008
Follow-up weight (kg)	Mean (SD) Median (IQR)	35.5 (14.3) 48 32.0 (25.1–42.6)	43.8 (17.1) 44 37.2 (33.6–55.2)	0.005
Follow-up height (m)	Mean (SD) Median (IQR)	1.34 (0.116) 41 1.37 (0.126–1.42)	1.46 (0.18) 41 1.46 (1.38–1.57)	0.0006
Follow-up cholesterol (mmol/L)	Mean (SD) Median (IQR)	7.34 (1.37) 48 7.2 (6.4–8.3)	5.82 (0.92) 53 5.8 (5.1–6.3)	1.484e-07
Follow-up HDL-C (mmol/L)	Mean (SD) Median (IQR)	1.42 (0.33) 43 1.4 (1.13–1.68)	1.38 (0.37) 43 1.37 (1.16–1.52)	0.47
Follow-up triglyceride (mmol/L)	Mean (SD) Median (IQR)	0.94 (0.54) 42 0.8 (0.6–1.2)	0.91 (0.38) 42 0.82 (0.68–1.01)	0.87
Follow-up LDL-C (mmol/L)	Mean (SD) Median (IQR)	5.47 (1.26) 45 5.3 (4.6–6.45)	4.06 (0.97) 41 4.02 (3.3–4.6)	1.316e-06

Age>10 years		No N=46	Yes N=65	P value (Mann-Whitney)
Age (years)	Mean (SD) Median (IQR)	12.8 (1.7) 46 12.9 (11.1–14.0)	12.5 (1.7) 65 12.2 (10.8–13.8)	0.35
Sex	% male	50.0% (23/46)	47.7% (31/65)	0.81 (χ^2^)
Ethnicity	% Caucasian	76.1 (35/46)	83.1 (54/65)	0.36 (χ^2^)
CHD in parent/first-degree relative	% yes	23.9% (11/46)	32.8% (21/64)	0.31 (χ^2^)
CHD in any relative	%yes	41.3% (19/46)	49.2% (32/65)	0.41 (χ^2^)
CHD onset age in relative	Mean (SD) Median (IQR)	44.2 (11.5) 22 46 (36–52)	41.8 (11.5) 35 40 (37–48)	0.46
Mutation status	% yes	56.8% (21/37)	71.2% (42/59)	0.15 (χ^2^)
Diag weight (kg)	Mean (SD) Median (IQR)	51.5 (13.1) 43 49.2 (41.9–59.4)	49.4 (15.4) 59 48.6 (38–56.4)	0.35
Diag height (m)	Mean (SD) Median (IQR)	1.58 (0.10) 39 1.56 (1.51–1.67)	1.54 (0.12) 52 1.54 (1.48–1.64)	0.23
Diag cholesterol (mmol/L)	Mean (SD) Median (IQR)	6.73 (1.37) 46 6.6 (5.8–7.5)	7.57 (1.38) 65 7.43 (6.8–8.3)	0.001
Diag HDL-C (mmol/L)	Mean (SD) Median (IQR)	1.42 (0.37) 42 1.35 (1.2–1.6)	1.41 (0.30) 63 1.4 (1.2–1.6)	0.81
Diag triglyceride (mmol/L)	Mean (SD) Median (IQR)	1.03 (0.58) 41 0.8 (0.7–1.2)	1.04 (0.62) 62 0.9 (0.6–1.3)	0.85
Diag LDL-C (mmol/L)	Mean (SD) Median (IQR)	4.76 (1.28) 44 4.65 (4–5.4)	5.64 (1.37) 62 5.5 (4.7–6.4)	0.0007
Follow-up weight (kg)	Mean (SD) Median (IQR)	55.4 (14.3) 44 52.9 (43.9–63.3)	58.8 (14.7) 64 57.1 (50.9–62.7)	0.22
Follow-up height (m)	Mean (SD) Median (IQR)	1.59 (0.10) 34 1.57 (1.53–1.65)	1.63 (0.11) 53 1.63 (1.55–1.70)	0.05
Follow-up cholesterol (mmol/L)	Mean (SD) Median (IQR)	6.52 (1.33) 46 6.3 (5.6–7.1)	5.55 (1.22) 65 5.3 (4.72–6.3)	0.0004
Follow-up HDL-C (mmol/L)	Mean (SD) Median (IQR)	1.44 (0.36) 45 1.43 (1.2–1.6)	1.37 (0.27) 57 1.38 (1.2–1.5)	0.36
Follow-up triglyceride (mmol/L)	Mean (SD) Median (IQR)	1.04 (0.49) 44 0.90 (0.7–1.30)	0.97 (0.50) 58 0.82 (0.6–1.2)	0.33
Follow-up LDL-C (mmol/L)	Mean (SD) Median (IQR)	4.55 (1.27) 44 4.3 (3.8–5.1)	3.82 (1.21) 55 3.6 (2.9–4.8)	0.006

CHD, coronary heart disease; HDL-C, high-density lipoprotein cholesterol; LDL-C, low-density lipoprotein cholesterol.

We also examined the possibility that the 15 registering centres that are purely paediatric (97 children) might be prescribing statin therapy at a different rate from the 13 clinics seeing both adults and children (135 children). After adjustment for age >10 years, the HR (95% CI) for being on a statin was 2.34 (1.32 to 4.15) for those seen at a paediatric clinic (p=0.004). To examine the relative contributions of these characteristics in determining statin use we carried out a Cox proportional hazards models for time to statin prescription with adjustment for age at diagnosis, and HRs (95% CIs) are presented in online supplementary table S2 and as a Kaplan-Meier curve by tertiles of LDL-C in figure 2. After adjustment for age at diagnosis, diagnostic cholesterol (1.52 (1.26 to 1.84) per 1 SD increase), LDL-C (1.59 (1.28 to 1.96) per 1 SD increase) and clinic type (HR 2.39 95% CI 1.57 to 3.65) were significantly associated with time to commencement of statin use (all p<0.0001).

**Figure 3:**
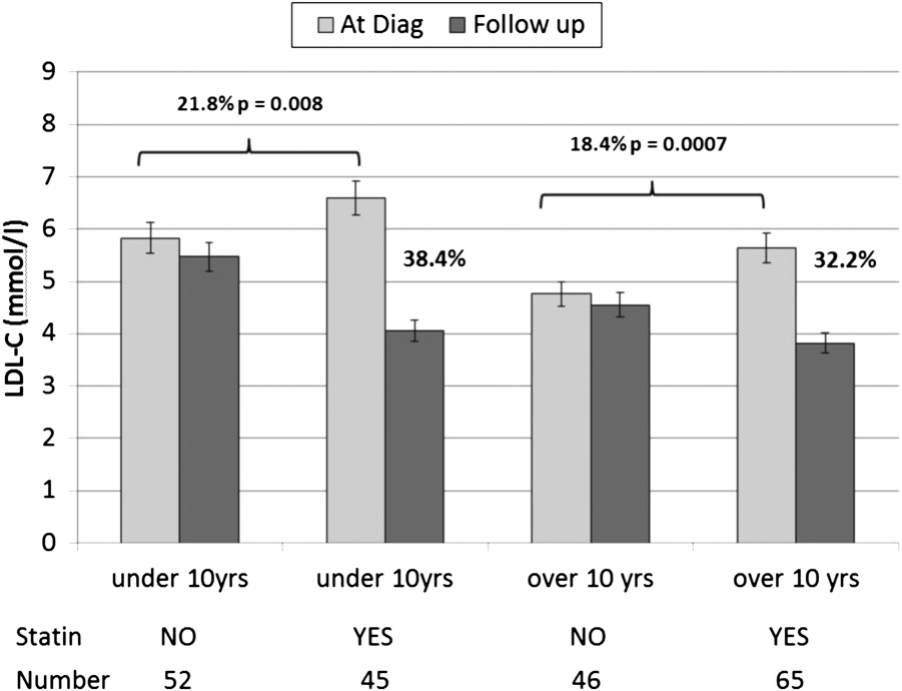
Change in low-density lipoprotein cholesterol (LDL-C) from baseline to follow-up value in children below and above the age of 10 years. The overall 5.4% reduction from LDL-C levels at diagnosis to follow-up in the children not being treated with statin may simply represent a regression to the mean effect, or possibly due to the effect of dietary fat restriction following dietician advice.

As shown in figure 3 in both age groups the diagnostic LDL-C in those who were subsequently treated was significantly higher than in those not currently on statins. Change in LDL with statin use did not differ between age groups (age by statin interaction, p=0.45). Overall, LDL-C levels in the statin-treated children fell by an average of 2.07 mmol/L (35%) achieving levels of 3.93 (1.11) mmol/L compared with 6.01 (1.46) mmol/L, a fall of 5.4% (0.29 (0.87) mmol/L) in the untreated group (p=1.2×10^−19^) (table 2 and figure 2). This difference remained after adjustment for diagnosis age and length of follow-up (estimated mean change (SE) 2.07 (0.12) for the treated group versus −0.25 (0.12) for the untreated group, p=1.1×10^−16^). However, there was a wide range in the fall of LDL-C levels from baseline, ranging from a 63% fall to a 15% rise in the statin-treated group, and from a 40% fall to a 50% rise in the untreated group. (For details on the statins prescribed see online supplementary table S2).

We addressed the issue of safety of statin use by analysing the measures of plasma creatine kinase (CK), alanine aminotransferase (ALT) and aspartate aminotransferase (AST) at follow-up. None of those on statin had measured plasma levels of CK, ALT and AST indicative of statin toxicity (ie, >2.5 times the normal range) (see online supplementary table 4 and figure S2).

## Discussion

The primary aim of this register is to collect baseline and long-term follow-up data on all children with FH in the UK, to see how well current NICE guidance on the diagnosis and management of children with HeFH is being adhered to, and to determine the safety and efficacy of lipid-lowering therapies, particularly when commenced below the age of 16 years. The children examined here have similar characteristics to the 147 children with data collected in the 2010 national FH audit,^21^ where the mean (IQR) LDL-C was 5.4 (4.5–6.1) mmol/L, HDL-C was 1.3 (1.1–1.6) mmol/L and TG 0.9 (0.7–1.2) mmol/L.^5^ It therefore appears that the children being entered onto the register are representative of those being seen in UK lipid clinics. Since essentially all of the registered children had been identified by family screening, the appropriate diagnostic LDL-C levels for HeFH are the NICE-recommended age and sex-specific cut-offs,^7^ and using these, at diagnosis, 95.3% had LDL-C levels above the age and sex cut-offs for being HeFH. Although NICE CG71 recommends DNA testing for all individuals with a diagnosis of HeFH; FH causing mutations were documented in 64% of children, the majority with a family mutation identified. While some families may not have consented to a genetic test, the reason for not performing a genetic test in children is primarily due to the lack of availability/funding of such tests in some parts of the country.

Since NICE recommends consideration of a statin for both boys and girls by the age of 10 years, the children were divided into those below 10 years (where only those with a poor family history of CHD or extreme LDL-C are expected to have been prescribed a statin) and those above 10 years when the majority would be expected to be on a statin. The data confirms that use of statins in early childhood and adolescence is primarily influenced by the age of the patient, the diagnostic level of TC and LDL-C, which are the major determinants of future risk of atherosclerosis, and of having a history of CHD in a parent or relative. Knowing the family mutation did not appear to influence commencing lipid-lowering drugs. Reassuringly, and as recommended by NICE CG71, girls were as likely as boys to have been started on statin therapy. Over 90% of those children prescribed a statin were taking a modest dose of 20 mgs or less, but reductions of LDL-C levels in the treated group were substantial (32–38%). Children often only require a low dose of statin to achieve a significant effect on LDL-C, compared with the sorts of doses (and potency) of statins being prescribed in adult FH clinics.^12–15^

There are currently no evidence-based recommendations on the optimal LDL-C reduction or level in children necessary to prevent the early development of significant atherosclerotic disease and subsequent cardiovascular events. The treated children demonstrated a mean 35% overall decrease in LDL-C but with a large range due presumably to different potency, compliance to treatment and dose of statin used. With the small number of patients currently available, a more detailed analysis is not possible. With respect to growth parameters, there was no significant difference in mean changes in cross-sectional weight and height in treated versus non-treated children, but a more appropriate analysis will be available when annual follow-up information is available. None of the children on statin treatment had evidence of statin toxicity as determined by measures of CK, ALT or AST.

One of the limitations of the Register is that by its nature it is an opportunistic sample and is likely to be biased to children with HeFH from more severely affected families than HeFH children in the general population. We also do not have data on the capture rate, recruitment rate, consent rate and retention for this register, or have details on any side effects of statin treatment such as muscle aches, and we are unaware of any similar FH children's register worldwide for other comparisons. We are currently working with the recruiting clinicians to ensure that as few as possible of the children are lost to follow-up as they transition to adulthood.

## References

[1] Nordestgaard BG, Chapman MJ, Humphries SE, et al Familial hypercholesterolaemia is underdiagnosed and undertreated in the general population: guidance for clinicians to prevent coronary heart disease: consensus statement of the European Atherosclerosis Society. Eur Heart J 2013;34:3478–90a. 10.1093/eurheartj/eht273 23956253 PMC3844152

[2] Benn M, Watts GF, Tybjaerg-Hansen A, et al Familial hypercholesterolemia in the danish general population: prevalence, coronary artery disease, and cholesterol-lowering medication. J Clin Endocrinol Metab 2012;97:3956–64. 10.1210/jc.2012-1563 22893714

[3] Do R, Stitziel NO, Won HH, et al Exome sequencing identifies rare LDLR and APOA5 alleles conferring risk for myocardial infarction. Nature 2015;518:102–6. 10.1038/nature13917 25487149 PMC4319990

[4] Marks D, Thorogood M, Neil HA, et al A review on the diagnosis, natural history, and treatment of familial hypercholesterolaemia. Atherosclerosis 2003;168:1–14. 10.1016/S0021-9150(02)00330-1 12732381

[5] Royal College of Physicians. Familial Hypecholesterolaemia Register and Audit. 2011 https://www.rcplondon.ac.uk/projects/familial-hypercholesterolemia-register-and-audit

[6] Wierzbicki AS, Humphries SE, Minhas R Familial hypercholesterolaemia: summary of NICE guidance. BMJ 2008;337:a1095 10.1136/bmj.a1095 18753174

[7] Starr B, Hadfield SG, Hutten BA, et al Development of sensitive and specific age- and gender-specific low-density lipoprotein cholesterol cutoffs for diagnosis of first-degree relatives with familial hypercholesterolaemia in cascade testing. Clin Chem Lab Med 2008;46:791–803. 10.1515/CCLM.2008.135 18601600

[8] Graham CA, McIlhatton BP, Kirk CW, et al Genetic screening protocol for familial hypercholesterolemia which includes splicing defects gives an improved mutation detection rate. Atherosclerosis 2005;182:331–40. 10.1016/j.atherosclerosis.2005.02.016 16159606

[9] Humphries SE, Whittall RA, Hubbart CS, et al Genetic causes of familial hypercholesterolaemia in patients in the UK: relation to plasma lipid levels and coronary heart disease risk. J Med Genet 2006;43:943–9. 10.1136/jmg.2006.038356 17142622 PMC2563208

[10] Talmud PJ, Shah S, Whittall R, et al Use of low-density lipoprotein cholesterol gene score to distinguish patients with polygenic and monogenic familial hypercholesterolaemia: a case-control study. Lancet 2013;381:1293–301. 10.1016/S0140-6736(12)62127-8 23433573

[11] Futema M, Shah S, Cooper JA, et al Refinement of variant selection for the LDL cholesterol genetic risk score in the diagnosis of the polygenic form of clinical familial hypercholesterolemia and replication in samples from 6 countries. Clin Chem 2015;61:231–8. 10.1373/clinchem.2014.231365 25414277 PMC4892342

[12] Goldberg AC, Hopkins PN, Toth PP, et al Familial hypercholesterolemia: screening, diagnosis and management of pediatric and adult patients: clinical guidance from The National Lipid Association Expert Panel on Familial Hypercholesterolemia. J Clin Lipidol 2011;5:S1–8. 10.1016/j.jacl.2011.04.003 21600525

[13] Descamps OS, Tenoutasse S, Stephenne X, et al Management of familial hypercholesterolemia in children and young adults: consensus paper developed by a panel of lipidologists, cardiologists, paediatricians, nutritionists, gastroenterologists, general practitioners and a patient organization. Atherosclerosis 2011;218:272–80. 10.1016/j.atherosclerosis.2011.06.016 21762914

[14] Watts GF, Sullivan DR, Poplawski N, et al Familial hypercholesterolaemia: a model of care for Australasia. Atheroscler Suppl 2011;12:221–63. 10.1016/j.atherosclerosissup.2011.06.001 21917530

[15] Harada-Shiba M, Arai H, Oikawa S, et al Guidelines for the management of familial hypercholesterolemia. J Atheroscler Thromb 2012;19:1043–60. 10.5551/jat.14621 23095242

[16] Vuorio A, Docherty KF, Humphries SE, et al Statin treatment of children with familial hypercholesterolemia—trying to balance incomplete evidence of long-term safety and clinical accountability: are we approaching a consensus? Atherosclerosis 2013;226:315–20. 10.1016/j.atherosclerosis.2012.10.032 23141908

[17] Vuorio A, Kuoppala J, Kovanen PT, et al Statins for children with familial hypercholesterolemia. Cochrane Database Syst Rev 2014;7:CD006401 10.1002/14651858.CD006401.pub3 25054950

[18] Narverud I, Retterstøl K, Iversen PO, et al Markers of atherosclerotic development in children with familial hypercholesterolemia: a literature review. Atherosclerosis 2014;235:299–309. 10.1016/j.atherosclerosis.2014.05.917 24908240

[19] Wiegman A, Hutten BA, de Groot E, et al Efficacy and safety of statin therapy in children with familial hypercholesterolemia: a randomized controlled trial. JAMA 2004;292:331–7. 10.1001/jama.292.3.331 15265847

[20] Kusters DM, Wiegman A, Kastelein JJ, et al Carotid intima-media thickness in children with familial hypercholesterolemia. Circ Res 2014;114:307–10. 10.1161/CIRCRESAHA.114.301430 24192652

[21] Seed M, Roughton M, Pedersen K, et al Current statin treatment, DNA testing and cascade testing of UK patients with FH. Prim Care Cardiovasc J 2012;5:181–5.

