# Supplementary material for: The UK Paediatric Familial Hypercholesterolaemia Register: preliminary data
Source: Arch Dis Child. 2016 Mar 6;102(3):255–60. doi: 10.1136/archdischild-2015-308570 (PMC5339547; doi:10.1136/archdischild-2015-308570)

Appendix 1

Development of the paediatric register: A steering committee was set up to develop the Paediatric register, including a patient representative, adult and child physicians and representatives of Heart UK, British Heart Foundation, British Inherited Metabolic Disease Group and the Royal College of Paediatrics and Child Health, all of whom endorsed the Register. A Clinical Champion was appointed in May 2012. The Register aims to collect information on the baseline characteristics of the children and to monitor the effects of current and new treatments on growth, puberty, routine biochemistry and to provide comparative audit and long term safety data, and anonymised data for research. Patient information sheets, age specific consents and assents, and parent information leaflets were developed and are available from the authors on request.

Contacting clinicians: To enrol patients to the Paediatric FH register, each centre was provided with a centre specific code and password. The local server and administrative team provided technical support. Clinical queries were addressed to the clinical champion. Newsletters and steering group meetings communicated updates and progress.

FH Paediatric Register Data Items

DEMOGRAPHIC
Site code
Patient audit no.
NHS Number
Hospital Number
Postcode
Patient Last name
Patient First name
Last edited
Dob
Gender
Consent
Clinic type
Ethnic group
Status (IN DATABASE)
DIAGNOSIS*
What is the patient's diagnosis?
Year of diagnosis?
What were the lipid and lipoprotein measurements used to make the diagnosis?
Height (cm)?
Weight (kg)?
Birth-weight
Gestational Age at birth
Does the patient have a history of CHD?
If yes this is substantiated by

- Angiogram
- Exercise ECG
- Other

Details of affected parent/relative (fill in only for affected relatives)
Does the child have the clinical sign of:

- Corneal arcus
- Tendon xanthoma

Has a family mutation has been found?
What is the family mutation, if known?
If the family mutation is known has the child been offered a DNA test?

Month and Year when offered

ASSESSMENT AND TREATMENT*

What was the date of the patient's last (most recent outpatient clinic appointment?

At the latest clinic what was the patient's height and weight?

What were the most recent lipid and lipoprotein measurements?

Patient on ANY lipid lowering treatment medication?

If patient is currently on lipid lowering treatment when was this commenced?

(If applicable) Please indicate the reason(s) that this patient is not receiving statin.

More than one option may be selected

- Patient intolerant to statin
- Patient/parent /carer declined
- Child under 10 years
- Child 10 and over but risk low
- Other, please list:

What was the patient's treatment at the time of the results?

- Statin:
- Atorvastatin Yes No Dose:
- Fluvastatin Yes No Dose:
- Pravastatin Yes No Dose:
- Rosuvastatin Yes No Dose:
- Simvastatin Yes No Dose:
- Patient no receiving statin:

Is the patient receiving any other lipid lowering treatment?

Only answer questions 2.7 (c-i) if answer to 2.6 is 'yes'.

- Resin
- Ezetimibe
- Fenofibrate
- Other, please list:

Plasma LDL-C apheresis

If the following were measured before initiation of statin treatment please provide levels:

- No measurements taken
- Date of measure
- creatine kinase
- ALT
- AST

If the following were measured after initiation of statin treatment please provide levels:

- No measurements taken
- Date of measure
- creatine kinase
- ALT
- AST

LIFESTYLE*

Is the patient a smoker?

If yes, age when they started?

If yes, how many a day do they smoke?

If the patient is female have they started menstruating?

If yes, age at first period?

If yes, are they taking an oral contraceptive?

Fields in Assessment and Treatment and Lifestyle REPEATED FOR EACH UPDATE*

Date of appointment

*Not available option for each question

In 2011, during the development of the Register, it was decided that detailed dietetic data would not be included as a data field, as the dietary management of FH is not standardised and would be beyond the scope of this register.

Appendix 2 – List of Centres Registering Data

Dr	Maurice	O'Kane	Altnagelvin Area Hospital
Dr	Saikat	Santra	Birmingham Children's Hospital
Dr	Graham	Bayly	Bristol Royal Infirmary
Dr	Paul	Masters	Chesterfield Royal Hospital
Dr	Peter	Sharpe	Craigavon Area Hospital
Dr	Ruth	Ayling	Derriford Hospital
Dr	Tim	Wang	Frimley Park Hospital
Dr	Gwyneth	Owen	Glangwili Hospital
Dr	Kok-Swee	Gan	Gloucestershire Royal Hospital
Prof	Tim	Aitman	Hammersmith Hospital
Dr	Mahmoud	Barbir	Harefield Hospital
Dr	Rochin	Patle	Medway Maritime Hospital
Dr	Steve	Jones	Pinderfields Hospital
Dr	Nirupa	D'Souza	Princess of Wales Hospital
Dr	Nigel	Capps	Princess Royal Hospital
Dr	Nicola	Pritchard	Royal Berkshire Hospital
Dr	Paul	Munyard	Royal Cornwall Hospital
Dr	D R	Nair	Royal Free London
Dr	Andrew	Taylor	Royal United Hospital Bath
Sister	Liz	Higginson	Russells Hall Hospital, Dudley
Dr	Inessa	Tracey	Sandwell General Hospital
Dr	Andar	Gunneberg	Swansea Hospital
Dr	Aabha	Sharma	Torbay Hospital
Dr	Yvette	Lolin	Tunbridge Wells Hospital
Dr	Peter	Dale	University Hospital of Wales
Dr	Nikki	Davis	University Hospital Southampton
Dr	Yee Ping	Teoh	Wrexham Maelor Hospital

Appendix 3 Supplementary Tables and Figures

Supplementary Figure 1. Correlation between Total and LDL-C measured at diagnosis and age, in those who subsequently did or did not receive statin treatment.

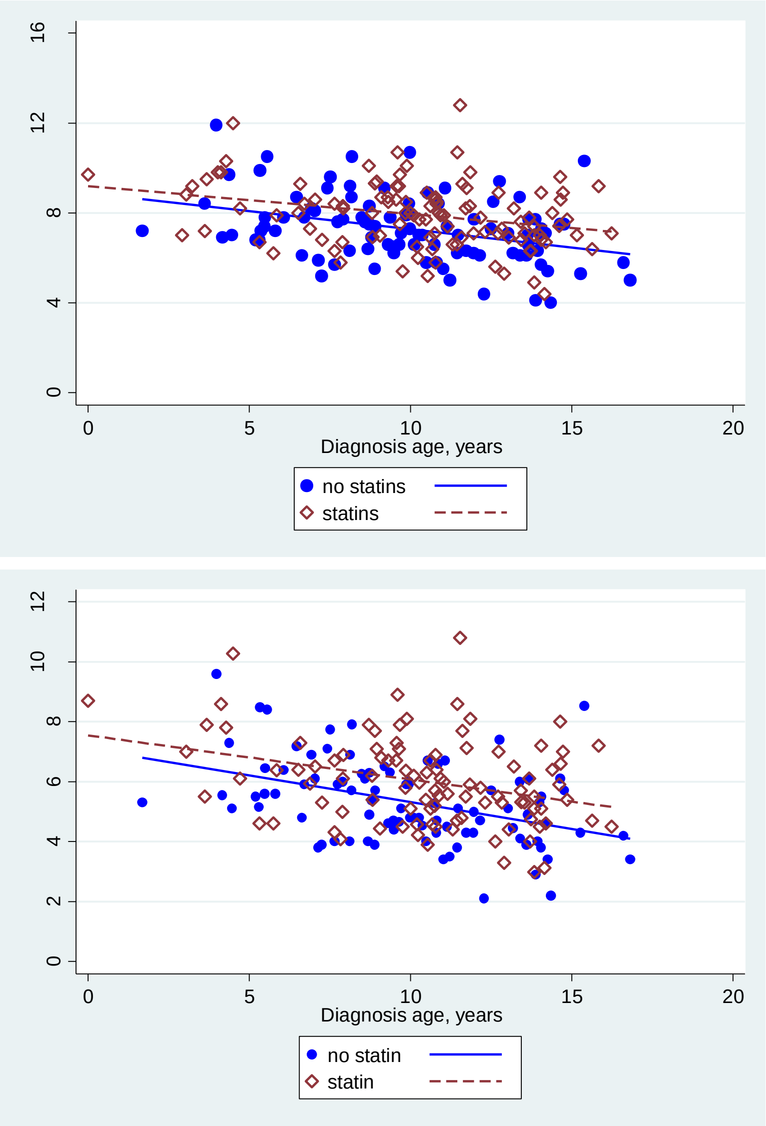

Supplementary Table 1 Correlation between diagnostic TC and age and diagnostic LDLC and age

	Association with TC	P value statin vs. no statin		Association with LDL-C	P value statin vs. no statin
No statin at FU	R=-0.36<br>B(se)= -0.16 (0.04)<br>P=0.0003	P=0.53	No statin at FU	R=-0.42<br>B(se)= -0.18 (0.04)<br>P=0.00003	P=0.60
Statins at FU	R=-0.29<br>B(se)= -0.12 (0.04)<br>P=0.002		Statin at FU	R=-0.32<br>B(se)= -0.15 (0.04)<br>P=0.001	
ALL	R=-0.30<br>B(se)= -0.14 (0.03)<br>P= 8.420e-06		ALL	R=-0.34<br>B(se)= -0.15 (0.03)<br>P= 1.417e-06	

Supplementary Table 2: Hazard ratios from Cox proportional hazards models for time to statin prescription with adjustment for age at diagnosis.

Variable		HR (95% CI) adjusted for age at diagnosis	P value	Number in model/Number on statins
Age (years)	1 year increase	1.16 (1.09-1.24)	3.0×10^{-6}	200/104
Sex	M:F	1.20 (0.81-1.78)	0.36	200/104
Ethnicity	Non Caucasian: Caucasian	0.84 (0.51-1.41)	0.51	200/104
CHD in parent/ first degree relative	Yes:No	1.06 (0.69-1.64)	0.78	198/102
CHD in any relative	Yes:No	1.05 (0.71-1.55)	0.82	205/108
Mutation status	Yes:No	1.23 (0.79-1.89)	0.36	179/97
Weight (kg)	Per 1 SD increase	0.76 (0.52-1.12)	0.17	183/93
Height (m)	Per 1 SD increase	0.68 (0.42-1.11)	0.12	164/83
Total Cholesterol (mmol/L)	Per 1 SD increase	1.52 (1.26-1.84)	0.00002	200/104
HDL-C (mmol/L)	Per 1 SD increase	1.04 (0.84-1.28)	0.73	186/97
Triglyceride (mmol/L)	Per 1 SD increase	1.08 (0.89-1.31)	0.44	183/96
LDL-C (mmol/L)	Per 1 SD increase	1.59 (1.28-1.96)	0.00002	186/95

This model excludes 5 who were on statins at diagnosis and 2 with missing dates for statin commencement.

Supplementary Table 3 Lipid Lowering treatment in Children with FH

Drug	% on drug (N)	Dose	% on dose(N)
Statin	47.8% (111)	5<br>10<br>20<br>30<br>40	3.6% (4)<br>63.1% (70)<br>27.0% (30)<br>1.8% (2)<br>4.5% (5)
	Atorvastatin 48.7% (54)<br>Pravastatin 28.8% (32)<br>Rosuvastatin 2.7% (3)<br>Simvastatin 19.8% (22)		
Resin-Cholestyramine	3.7% (4)	4<br>8<br>12	50.0% (2)<br>25.0% (1)<br>25.0% (1)
Ezetimibe	0.9% (1)	10	100% (1)
Fenofibrate	0 (0)	-	-
other (Benecol, plant stannol)	1.8% (2)	-	-

The commonly used statins were atorvastatin (48.7%; n=54; pravastatin (28.8%, n=32); rosuvastatin (2.7%; n=3) and simvastatin (19.8%; n=22). There was no significant difference in age at treatment (p=0.28) in those on different statins (means (ranges) for age at last visit; pravastatin 12.9 (7-16), atorvastatin 14.0 (2-20) and simvastatin 13.6 (4-19). A small proportion of patients were on resins (<5%) and one patient was reported to be on ezetimibe. No patients were on fibrates and the use of plant stanols was limited (1.8%, n=2).

Supplementary Table 4 Markers of statin damage by statin use

Follow up levels		No N=121	Yes N=111	P value (Mann-Whitney)
Creatine Kinase (CK) U/L;	Mean (SD)<br>Median [IQR]	144 (33.9) 2<br>144 [120-168]	123.3 (56.2) 76<br>107 [86.5-147.5]	0.33
Alanine Amino Transferase (ALT) U/L	Mean (SD)<br>Median [IQR]	14.3 (4.8) 4<br>14 [10.5-18]	19.7 (8.3) 101<br>17 [14-22]	0.15
Aspartate Amino Transferase (AST) U/L	Mean (SD)<br>Median [IQR]	25 (6.7) 4<br>23.5 [20-30]	25.6 (5.8) 26<br>26.5 [22-29]	0.74

For toxicity reporting in paediatric studies, the Common Terminologies Criteria for Adverse Events (CTCAE) guidelines are used, which do not discriminate between paediatric and adult subjects and determine the toxicity level of ALT/AST and CK rise as a factor of ULN (CTCAE guidelines, 2003). CTCAE grading of toxicity for LFTs is defined as follows: Grade 1 values > 2.5 x Upper Limit of Normal (ULN); Grade 2: < 2.5 to 5 x ULN; grade 3 > 5 to 20 x ULN; Grade 4 > 20 x ULN; Grade 5 death. Some paediatric studies have used ALT/ALT > 250 IU/L as criteria for toxicity. CPK: grade 1 2.5 x ULN; Grade 2 >2. 5 x ULN; Grade 3 > 5 x ULN; Grade 4 > 10 x ULN; Grade 5 death.

Cancer Therapy Evaluation Program, Common Terminology Criteria for Adverse Events, Version 3.0, DCTD, NCI, NIH, DHHS March 31, 2003 (http://ctep.cancer.gov), Publish Date: August 9, 2006

Supplementary Figure 2

AST level (N=26) post statin treatment

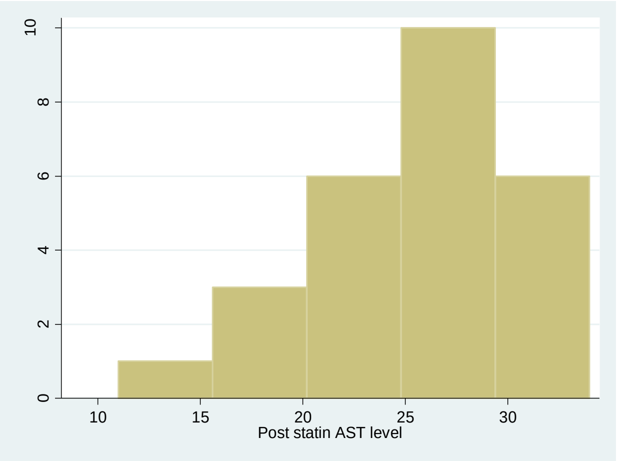

ALT levels post statin treatment (n=101)

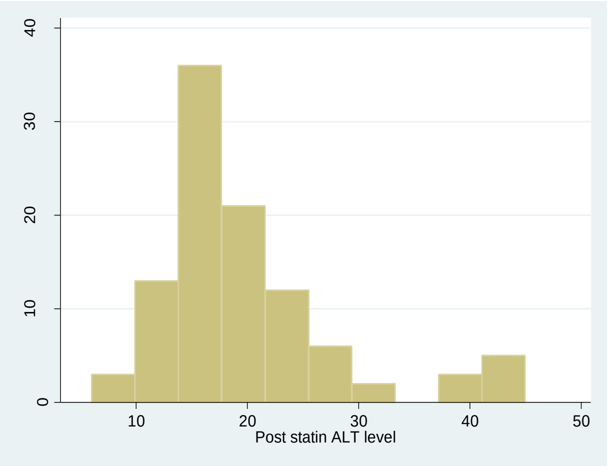

Creatine Kinase levels post statin treatment (n=76)

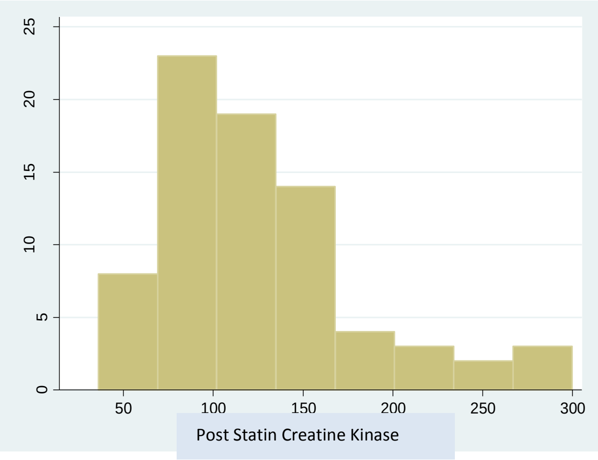

Supplement: Supplementary appendix [file archdischild-2015-308570supp.pdf]
